# Testing Biological and Psychological Pathways of Emotion Regulation as a Primary Mechanism of Action in Yoga Interventions for Chronic Low Back Pain: Protocol for a Randomized Controlled Trial

**DOI:** 10.2196/56016

**Published:** 2024-03-14

**Authors:** Angela R Starkweather, Wanli Xu, Katherine E Gnall, Mariel Emrich, Camille L Garnsey, Zachary E Magin, Weizi Wu, Joseph Fetta, Erik J Groessl, Crystal Park

**Affiliations:** 1 Department of Biobehavioral Nursing Science College of Nursing University of Florida Gainesville, FL United States; 2 School of Nursing University of Connecticut Storrs, CT United States; 3 Department of Psychological Sciences University of Connecticut Storrs, CT United States; 4 Herbert Wertheim School of Public Health & Human Longevity Science University of California San Diego San Diego, CA United States; 5 Veteran's Affairs San Diego Health System San Diego, CA United States

**Keywords:** chronic low back pain, clinical trial, emotion regulation, mind-body interventions, pain sensitivity, yoga

## Abstract

**Background:**

Interventions that promote adaptive emotion regulation (ER) skills reduce pain in patients with chronic pain; however, whether the effects of yoga practice on chronic low back pain (CLBP) are due to improvements in ER remains to be examined.

**Objective:**

This study will test whether the effects of yoga on CLBP (improved pain severity and interference) are mediated by improved ER, the extent to which effects are related to specific aspects of ER, and the role of pain sensitization as a mediator or moderator of effects. In this study, pain sensitization will be assessed by quantitative sensory testing and gene expression profiles to examine whether pain sensitization moderates yoga’s effects on pain or whether yoga and ER abilities reduce pain sensitization, leading to decreased pain severity and interference.

**Methods:**

For this 2-arm parallel group blinded randomized controlled trial, we will enroll 204 adults with CLBP who will be randomized to receive the yoga (n=102) or a control stretching and strengthening (n=102) intervention, which are delivered via web-based synchronous biweekly 75-minute sessions over 12 weeks. Participants are encouraged to practice postures or exercises for 25 minutes on other days using accessible prerecorded practice videos that are sent to participants digitally. Participants will be assessed at 5 time points: baseline, midintervention (6 weeks), postintervention (12 weeks), and 3- and 6-month follow-ups. Assessments of ER, pain severity and interference, pain sensitivity including somatosensory and gene expression profiles, and physical strength and flexibility will be conducted at each visit. The fidelity of the interventions is assessed using a manualized checklist to evaluate recorded group sessions to ensure consistent instructor delivery.

**Results:**

The primary outcome will be the mean change in pain severity as measured by the Brief Pain Inventory-Short Form at 12 weeks. The primary mechanism of action is ER measured by change in the Difficulties in Emotion Regulation Scale total score. Secondary outcomes include pain sensitivity, physical strength and flexibility, pain interference, and quality of life. A mediation path analysis and series of moderated mediation path analyses will be conducted to test the study hypotheses. As of January 2024, we have enrolled 138 participants. We expect the study to be completed by May 2025.

**Conclusions:**

The study will provide important data for evaluating whether improvements in ER are responsible for reduced pain perception and pain sensitivity as well as increased quality of life in the context of chronic pain. The study findings have important implications for determining the mechanism of action for yoga and possibly other mind-body interventions as nonpharmacological therapies for pain management. The results of the study will inform the content, delivery, and measures for intervention trials involving yoga as a modality for relieving pain and improving function.

**Trial Registration:**

ClinicalTrials.gov NCT04678297; https://clinicaltrials.gov/study/NCT04678297

**International Registered Report Identifier (IRRID):**

DERR1-10.2196/56016

## Introduction

### Background

Yoga has been shown to reduce pain and improve function in populations with chronic low back pain (CLBP) in multiple randomized controlled trials (RCTs) [1,2]. However, results vary, and effects are generally modest [3]. This variation is perhaps due to differences in the specific neurobiological mechanisms through which yoga exerts clinical improvements on pain that are affected by different yoga interventions. These mechanisms, which have been only minimally identified, could potentially be harnessed to achieve more consistent and optimal outcomes from yoga interventions. A study that examined mediators of yoga’s effects on pain lacked strong theoretical bases for assessment of the mechanisms involved [4]. Using a comprehensive theoretical emotion regulation (ER) model of yoga [5], we will conduct the first test of ER as a primary mechanism of yoga’s effects on CLBP. We will also test the role of pain sensitization using somatosensory and gene expression profiles in this model [6] and examine the specific aspects of ER.

ER refers to “extrinsic and intrinsic processes responsible for monitoring, evaluating, and modifying emotional reactions, especially their intensive and temporal features, to accomplish one’s goals” [7]. Emotions strongly influence perceptions of pain intensity and predict disability [8], particularly among individuals with CLBP [9,10]. Interventions that promote adaptive ER skills reduce pain in patients with chronic pain [11]. Increasing evidence demonstrates that consistent yoga practice can promote improved ER [12,13], but research has not yet tested whether the effects of yoga practice on CLBP are due to improvements in ER.

### Integrative Model of Yoga’s Effects on ER

Given the importance of ER in managing chronic pain and yoga’s demonstrated promotion of improved ER, we developed an integrative model of yoga’s effects on CLBP that highlights the key role of ER. Previous work by our team [14,15] and others [16] demonstrated that yoga simultaneously comprises multiple components and that physical activity (poses and transitions between poses), breathwork, and meditation are all important therapeutic elements. Thus, although physical activity interventions alone have demonstrated salutary effects on negative emotion [17] and ER [18,19], yoga’s inclusion of breathwork and meditation along with physical activity may more potently improve ER [5].

**Figure 1:**
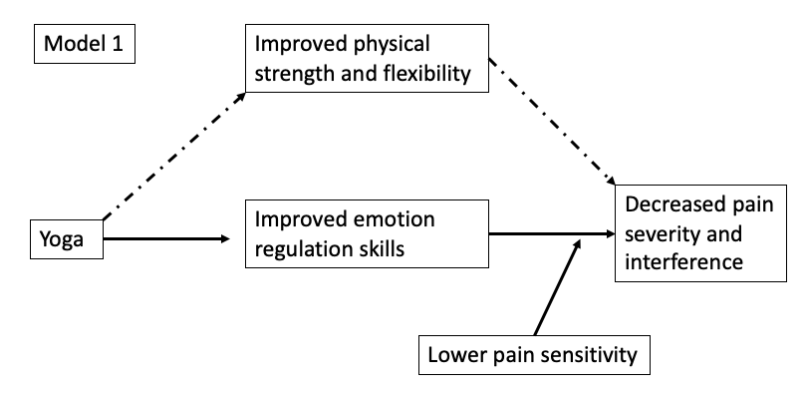
Model of primary and alternative mechanisms of action. Model 1 proposes that pain sensitization moderates yoga’s effects on pain, in that chronic low back pain patients with higher sensitization may benefit less given that their pain is more centralized.

Yoga provides myriad opportunities to develop ER skills helpful to people managing chronic pain. Practitioners can learn how to experience sensation without negative evaluative judgments, cultivating nonreactivity. Due to cognitive priming and reinforcement during movement, yoga provides simultaneous input to cognitive, motor, and sensory pathways, which may allow practitioners to consciously influence their interpretation of sensation or pain and manage its affective dimensions. Given the myriad components of yoga that can facilitate developing ER skills, we posit that ER is a key mechanism by which yoga exerts effects on individuals with CLBP. The few studies examining links among yoga, ER, and pain support our model (Figure 1). For example, yoga practitioners were better able to tolerate experimentally induced pain compared to nonpractitioners, with corresponding greater left intrainsular white matter connectivity [20]. Further, the yoga practitioners reported coping with the pain stimulus using (adaptive) ER strategies such as relaxation, nonjudgmental focusing, and reappraisal. Thus, the ability to minimize the affective dimensions of pain may dampen physiological responses to negative emotions such as anxiety, which have been associated with the perception of pain [21,22].

### Pain Sensitivity May Influence the Effects of Yoga and ER on CLBP via Moderation or Mediation

Recent studies document that patients with CLBP demonstrate sensory and functional alterations reflecting peripheral and central nervous system sensitization associated with enhanced pain sensitivity [23-25]. Enhanced pain sensitivity as a mechanism of CLBP includes sensitization of nociceptors and neuronal circuits [26] and increased pain signaling through membrane excitability and synaptic efficacy [27], which are conferred through altered expression of pain sensitivity (and immune-related) genes [6,28]. The somatosensory changes associated with enhanced pain sensitivity in CLBP involve decreased thermal and mechanical pain thresholds at the site of pain and remote areas [25,26].

Preliminary research suggests that negative emotion is an important determinant of pain sensitivity [29,30], predicting up to 23% of the variance in CLBP-related disability [31,32]. For instance, greater pain catastrophizing and anxiety are associated with lower standardized values of heat pain threshold and tolerance in patients with chronic pain [33]. In turn, negative emotions and inability to tolerate stress have been shown to activate inflammatory pathways through the release of proinflammatory cytokines and substance P [34,35]. To date, only 2 small studies have examined whether yoga specifically influences pain sensitivity. However, in a systematic review of 15 RCTs, yoga was associated with decreases in interleukin-1β, interleukin-6, and tumor necrosis factor-α [36]. In addition, specific components of yoga (meditation, relaxation, and breathing) have been shown to alter gene expression profiles and levels of inflammatory mediators [37-39].

**Figure 2:**
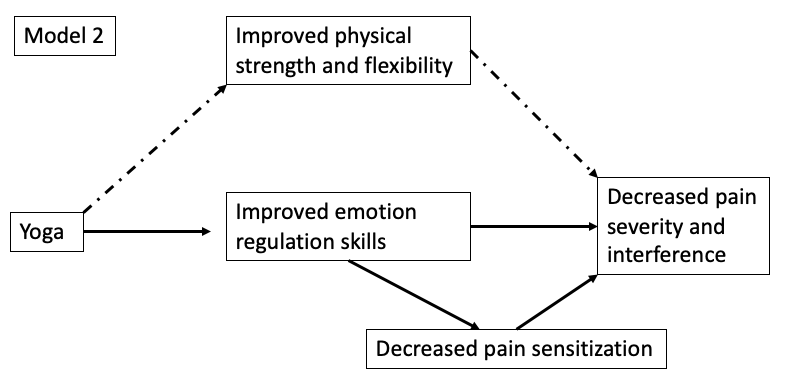
Moderated mediation model of emotion regulation. Model 2 will explore the possibility that yoga and improved emotion regulation abilities might actually improve sensitization and thus decrease pain.

As shown in Figure 1, we propose that pain sensitization moderates yoga’s effects on pain, where patients with CLBP with higher sensitization may benefit less [40,41], given that their pain is more centralized [42,43]. This is a fairly novel hypothesis with some supportive evidence [44,45]. We will also explore the possibility that yoga and improved ER abilities might actually *improve* sensitization and thus decrease pain (Figure 2).

Little research has demonstrated that treatments for pain, including behavioral interventions, modify sensitization once it has occurred [46,47], although a reversal of central sensitization was observed in patients with structural pain–related pathology who underwent corrective surgery [48] as well as patients with migraine and chronic pancreatitis [49,50]. We considered alternative strategies to test these linkages. For example, a systematic review of 1037 individuals with CLBP provided moderate evidence that structural brain differences in specific cortical and subcortical areas of the brain and altered functional connectivity in pain-related areas following painful stimulation differentiate chronic pain from pain-free individuals [51]. While brain imaging provides 1 way to examine changes in pain sensitivity, we assert that identifying specific cellular mechanisms through which ER affects pain sensitivity will generate greater translational capacity for the clinical care of individuals with CLBP.

### Controlling for Improvements in Physical Strength and Flexibility

To be comprehensive, our model includes improved strength and flexibility, given that improvements in these domains are common in yoga intervention trials. In fact, yoga typically shows the effects on strength, flexibility, and other physical outcomes that are as substantial as those of physical therapy or stretching-strengthening exercise groups [52,53]. Sherman et al [4] demonstrated that strength and flexibility may mediate some of yoga’s effects on pain. Overall, accumulating evidence demonstrates moderate but varied effects of yoga for reducing CLBP pain and disability; much remains to be learned about *how* yoga exerts these effects and why its effects vary. Understanding the proposed mechanism of actions (MOAs) of yoga will provide not only a more solid scientific basis to assure clinicians of the value of yoga to patients with CLBP and other conditions but also important targets for intervention modification and optimization.

### Objectives

To address this gap, we will test the impact of a yoga intervention on ER and pain severity and functioning compared to a general stretching or strengthening intervention. This design allows us to isolate the additional effects of yoga above and beyond more general movement practices, thereby testing ER as a key MOA underlying the clinical effects of yoga on CLBP. Specifically, we will test whether yoga’s effects on CLBP (improved pain severity and interference) are mediated by improved ER, the extent to which effects are related to specific aspects of ER, and the role of pain sensitization as a mediator or moderator of effects.

For the active comparison intervention, we chose to use stretching and strengthening exercises, given that we are interested in determining not only whether yoga influences ER but also the extent to which these effects are due to aspects of yoga above and beyond nonspecific factors (eg, expectancies, time, attention, and effort) [13] as well as improvements in strength and flexibility [4]. The 2 intervention groups will be similar in many aspects, but only yoga will incorporate aspects that we hypothesize will facilitate ER (eg, breathwork, mindfulness, and relaxation). We selected the hypothesized MOA based on our extensive work regarding the therapeutic characteristics of yoga and mechanisms of CLBP [5,14]. Our 3-month and 6-month follow-ups will allow us to examine whether the effects of yoga emerge later or persist. The study is highly innovative in its approach to testing the MOA of yoga for alleviating CLBP. By identifying the specific mechanisms through which yoga may influence pain, these results will facilitate optimizing interventions to improve therapeutic accuracy for prescribing and using yoga for CLBP, a highly prevalent and costly health condition in the United States and worldwide.

## Methods

### Design

**Figure 3:**
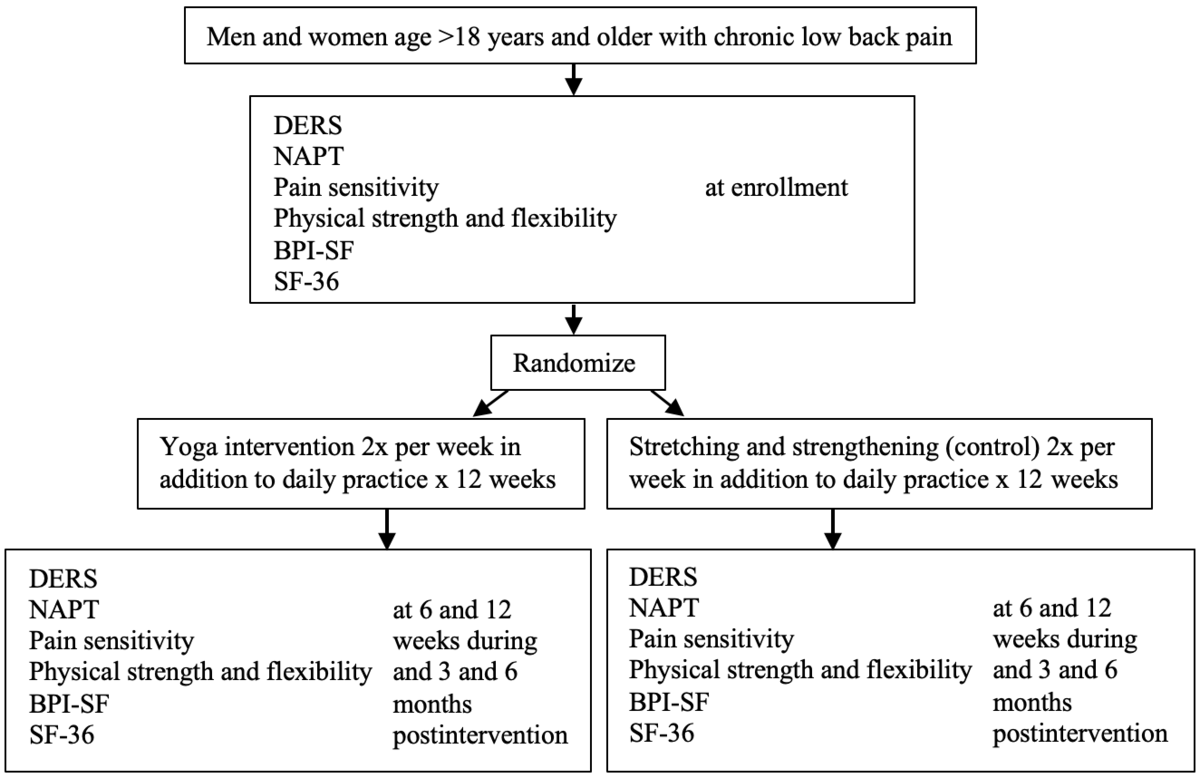
Study overview. BPI-SF: Brief Pain Inventory Short-form; DERS: Difficulties with Emotion Regulation Scale; NAPT: Negative Affective Priming Task; Pain sensitivity measured by quantitative sensory testing and blood drawn for gene expression profile; SF-36: 36-item Short Form Health Survey.

This study is a 2-arm parallel group blinded RCT to examine ER as a MOA of yoga in individuals with CLBP (Figure 3). In total, 204 people with CLBP will be randomized to receive yoga or the stretching and strengthening intervention with assessments of pain severity and interference; ER; pain sensitivity; physical strength and flexibility; and quality of life at baseline, 6 weeks (midway through the intervention), 12 weeks (after completion of the intervention), and 3 and 6 months after the completion of the intervention. Both groups will be instructed to continue the use of any other strategies or treatments that they have been using to manage their CLBP throughout the study.

### Participants

We will enroll 204 men and women aged 18 years and older with nonspecific CLBP who meet eligibility (Textbox 1). Nonspecific CLBP is defined as pain without a specific cause or need for surgical intervention that is anywhere in the region of the low back bound superiorly by the thoracolumbar junction and inferiorly by the lumbosacral junction, which has been present for more than 3 months out of the prior 6 months and is currently rated at a level of ≥2 on the numeric rating scale. Eligibility will be determined by asking the following screening questions [54]: (1) How long has back pain been an ongoing problem for you? (2) How often has low-back pain been an ongoing problem for you over the past 6 months? Responses of greater than 3 months to question 1 and “at least half the days in the past 6 months” to question 2 will define CLBP.

**Inclusion and exclusion criteria to determine eligibility for study participation.**

**Inclusion criteria**

- Aged 18 years and older
- Report low back pain for more than 3 months out of 6 months
- Willing to attend 12 weeks of yoga or stretching (twice per week)
- Willing to complete 5 assessments
- English literacy
- No changes in pain treatments in the past month
- Willing to not change pain treatments during the study unless medically necessary
- Have not practiced yoga more than 2 times in the last 3 months

**Exclusion criteria**

- Back surgery within the last 1 year
- Back pain due to specific systemic problem (eg, lupus)
- Lower extremity weakness (motor strength four-fifths of the quads, glutes, hamstrings, and extensor hallucis longus)
- Sciatica or (+) straight leg raise
- Coexisting chronic pain problem (migraine headaches and fibromyalgia)
- Serious or unstable psychiatric illness (eg, psychosis, mania, and history of suicide attempt) and current suicidal ideation
- Major coexisting medical illness (eg, cancer, chronic obstructive pulmonary disease, and morbid obesity)
- Positive Romberg test (with or without sensory neuropathy)

### Recruitment and Retention Strategies

Active and passive recruitment strategies will be used to recruit individuals with CLBP include (1) contacting CLBP participants enrolled in an institutional review board (IRB)–approved pain registry, (2) advertisements on social media (Instagram, Facebook, Twitter, Reddit, and Craigslist), (3) placing printed advertisements in public transportation vehicles and local newspapers, and (4) distributing flyers at local outpatient clinical sites and local universities. We will recruit continuously and start a new cohort every 6 months, so that there will be cohorts in both interventions and follow-up phases throughout the study. This approach will help control for seasonal effects and other potential confounds. All participants will receive a yoga or exercise mat, block, and strap prior to their first session, and US $70 following the baseline, 6- and 12-week postintervention, and 3- and 6-month follow-up assessments with an additional US $50 for those who complete all data collection sessions.

Interested volunteers are asked to complete a web-based screening questionnaire via REDCap (Research Electronic Data Capture; Vanderbilt University), which includes questions to ensure they meet inclusion and exclusion criteria and can participate safely. Volunteers who are deemed eligible to participate are contacted by study staff to schedule their baseline appointment.

### Ethical Considerations

The study was approved by the University of Connecticut IRB (#H19-191). Prior to completing any study procedures, all participants provide written informed consent on a form that was approved by the IRB at the University of Connecticut (IRB#H19-191). All study procedures are in accordance with the ethical standards described by the Helsinki Declaration. Study participants are assigned an identification number, allowing all stored records to be deidentified. Deidentified data are kept in locked file room or stored in a password-protected database on a password-protected computer. All participants are monetarily compensated for their participation up to US $400 for completing all study visits. Participants can withdraw from the study at any time.

### Procedures

#### Randomization and Blinding

Eligible volunteers are scheduled to meet with a study staff member who will follow all informed consent procedures. After written consent is obtained, participants are randomized to the yoga or stretching and strengthening group using a stratified randomization scheme based on biological sex to ensure balance between groups, and the assigned group is known only by the study staff managing the participant and instructor schedules.

We will use blinding whenever possible. Assessments, evaluations, and data analysis will be conducted without the knowledge of participants’ assigned group. We will blind the study personnel to the assigned condition of subjects when conducting data collection by using unique study identification numbers, by following a strict script to refrain from discussing participant activities, and by using different members of the study team to coordinate assigned condition activities and collect data. Deidentified data with codes for assigned condition will facilitate blinded data analysis.

#### Data Collection Visits

At each study visit, venous blood samples (15 mL) will be collected in one 10-mL PAXgene vacutainer and one 6-mL K2 ethylenediaminetetraacetic acid vacutainer by a research team member experienced in venipuncture. The samples will be immediately placed in a biohazard container and transported to the laboratory where they will be processed and stored in a –80 °C freezer for bulk processing of genetic and protein measures. Physiological and quantitative sensory measurements will be conducted by a trained research assistant. Participants will be asked to complete all study questionnaires via REDCap after completion of the laboratory data collection.

#### Interventions

All yoga and stretching and strengthening sessions will take place on a secure web-based platform. This delivery modality will provide more inclusive participation and reduce barriers such as participant transportation and parking. For both study arms, 75-minute classes will be offered 3 times per week for 12 weeks. Participants will be asked to attend at least 2 out of 3 of the offered classes per week. The importance of attending 2 sessions per week is emphasized during the first session and throughout all subsequent sessions. Participants who repeatedly miss sessions without notifying the study personnel will be contacted to check on the reason for nonattendance and to encourage resuming participation as soon as possible. Participants are encouraged to consult with the instructor individually before or after class about problems or difficulties they are encountering at home or in the formal sessions.

Participants are asked to practice exercises at home on days they do not attend formal synchronous sessions to maximize the benefit of the intervention. Participants will be emailed daily links to prerecorded practice videos that are approximately 25 minutes in length and asked to record the amount of time they spent on the practice videos.

#### Yoga Intervention

##### Overview

The yoga intervention will be led by certified 500-hour registered yoga teachers trained in working with patients with back pain. The yoga for CLBP protocol [55] consists of classical hatha yoga with influences from vinyasa and Iyengar yoga. These yoga styles are those most commonly offered in clinical and community settings and emphasize the importance of modifications and adaptations including the use of props such as straps and blocks to minimize the risk of injury and make the poses accessible to people with health problems and limitations [56]. The manual developed to standardize the yoga classes offered in the intervention contains pictures and a description of how to perform each pose. The manual also includes suggested instructor dialogue, which may be paraphrased or varied slightly yet promotes consistent delivery, which enhances the replicability and generalization of results. The intervention includes some poses that could aggravate back conditions in some people if they are not modified; therefore, multiple modifications are suggested, and many poses are only worked up to after weeks of practice and individual attention from the instructor. The more challenging poses are never included in the home practice videos.

For each yoga class in the protocol, the instructor leads participants through a series of 23 yoga poses (32 total variations) at a slow-moderate pace. During the formal sessions, participants are instructed to take slow deep breaths, timing their inhales and their exhales with specific phases of poses. Participants are encouraged to emulate optimal alignment as demonstrated by the instructor and to focus on a goal or positive direction for their yoga practice. Yoga classes are constructed to allow optimal flow from one pose to another. Each session begins with a few minutes of deep breathing and mindfulness or meditation followed by 15 minutes of basic postures (poses 1-8) to warm up muscles by increasing circulation to provide increased flexibility as the class progresses. Most poses are conducted once per side. After the warm up, the instructor leads participants through a series of standing poses (poses 9-14) for 15 minutes. After the standing poses, the class moves into floor poses (poses 15-23) for 20 minutes. Sessions end with 5-7 minutes of complete relaxation in the standard ending pose “savasana,” during which additional positive affirmations are provided.

##### Stretching and Strengthening Control Intervention

This intervention is designed to require a similar amount of physical exertion (stretching and strengthening exercises only, with no extreme movement). Classes will be led by physical therapists or other skilled therapeutic exercise instructors and involve conventional exercises appropriate for patients with CLBP, including a comprehensive set of exercises that stretch all the major muscle groups, with an emphasis on the trunk and legs. The intervention will include 12 stretching exercises used in the exercise arm of previous studies that have successfully compared exercise to yoga [57,58] (ie, gastrocnemius, soleus, quadriceps, posterior and inferior shoulder, upper trapezius, hip flexor, back extension, back rotation, hamstrings, hip external rotators, and back flexion). It will also include 3 additional stretches (hip internal rotators, hip adductors, and hip flexion). Each stretch will be held for approximately 60 seconds and repeated once. In addition to a complete set of full-body stretches, the class will begin with a 5-minute warm up period consisting of basic aerobics steps (ie, 1 minute each of walking in place, marching, lateral shuffling, turning and reaching, and box step) and will also include 4 exercises that strengthen the back, abdomen, and hips (ie, squats, crunches, oblique crunches, and back extensions). Over the 12 weeks, the repetitions of each strength exercise are increased from 8 to 30. Specific strength exercises are practiced in separate sets of 5 to 10 repetitions.

#### Treatment Fidelity

All sessions will be recorded, and instructor fidelity to the yoga intervention or stretching intervention manuals will be evaluated using video-recordings of sessions. Instructors and participants will be aware of and consent to the recordings. We will select 1 session per week from each condition for rating by trained research assistants, who will review sessions using a manual checklist for instructor adherence to the manuals, using a 0-10 rating scale for each procedure or instruction. If the rating is less than 100% adherent, we will meet with the instructor to discuss the places in the session that were not adherent.

### Measures

The assessments of pain intensity, pain interference, ER, and other parameters will be taken at baseline, 6 and 12 weeks, and 3 and 6 months post intervention.

#### Primary Outcome: Primary MOA

Pain severity will be measured with the Brief Pain Inventory-Short Form (BPI-SF) [59] at 12 weeks. The BPI-SF has been validated with CLBP and has shown good reliability [60].

ER as the primary MOA will be measured using the Difficulties in Emotion Regulation Scale (DERS) score at 12 weeks. The DERS is the gold standard self-report measure of individuals’ abilities to respond to emotional experiences in a goal-oriented manner [60]. The 36-item DERS taps aspects of difficulties with ER, which we anticipate will lessen with yoga practice [61]. Participants rate the extent to which each of 36 statements currently applies to them, and the measure yields a total score comprising summed subscales. The DERS is sensitive to change over time in ER, and internal consistency reliability is high [60].

The Negative Affective Priming Task [62] will also be used to capture individual differences in emotional attention, reactivity and appraisal, and ability to inhibit negative emotion and purposefully shift attention to more positive aspects of daily life. Participants are exposed to neutral and affective words with a strong positive and negative valence in consecutive paired trials, one word serving as the “target” and the other as a “distractor.” Participants are instructed to ignore the distractor and to attend to the target in both control and negative priming conditions, with “inhibition ability” operationalized as a difference score between response latencies in control and negative priming conditions. The stronger one’s inhibition abilities, the longer the latency in the negative priming condition compared to the control condition (scored in milliseconds). Individuals with chronic depression and greater tendency to engage in maladaptive rumination demonstrate reduced ability to inhibit negative, as opposed to neutral, words with no difference in the ability to inhibit positive words [63,64].

#### Secondary Outcomes

##### Overview

There are 4 secondary outcomes for this study: pain sensitivity, physical strength and flexibility, pain interference, and quality of life.

##### Pain Sensitivity: Peripheral and Central Sensitivity

We will use a comprehensive quantitative sensory testing (QST) battery of mechanical and thermal stimuli [65]: (1) cutaneous mechanical pain sensitivity, involving measures of tolerance, threshold, temporal summation, and after sensations; (2) heat and cold pain sensitivity, involving threshold, tolerance, and ratings of suprathreshold stimuli; and (3) pressure pain thresholds. Interrater reliability of the QST protocol has been reported in several clinical trials, with each individual test achieving a CI of 0.87-0.94 [66,67].

In addition to the QST battery, we will isolate total RNA from whole blood for sequencing at each time point. We will prepare libraries per Illumina standard protocols and sequence RNA on the Illumina HiSeq2500 System (Illumina Inc) to obtain 150 base pair paired-end reads. We will examine how changes in the expression of pain sensitivity genes, as well as the entire transcriptome, differ between the experimental and control group (those with complete data from yoga vs stretching) × sex using statistical modeling and construct a database of gene expression profiles to generate a profile of change in ER, as well as identify novel therapeutic targets for better pain management. We will use a candidate approach to examine genes identified from our preliminary studies, and a hypothesis-generating approach to identify differentially expressed genes.

##### Physical Strength and Flexibility

Physical measures include grip strength and core stabilization and strength. Grip strength will be measured using 2 trials for each hand with a hydraulic dynamometer. The best performance is selected for each side, and the average of the left and right hand is used for analysis. Painful or injured hands or wrists are not tested, and the result of the good hand is used. Good reliability and predictive validity have been shown [68]. Core stabilization and strength will be assessed using prone and supine bridge positions [69]. Participants begin on their elbows in the prone position with shoulders, hips, and ankles aligned. The supine position is tested next, with knees flexed 90° and pelvis raised from the floor with shoulders, hips, and knees aligned. Assessors record the length of time (120 seconds maximum) that each position is held in proper form.

##### Pain Interference

Interference of function due to pain will be measured with the Brief Pain Inventory [60]. The pain interference score is the mean of the 7 interference items.

##### Quality of Life

The 36-item Short Form Health Survey [70] will be administered at each time point to gain a deeper understanding of the psychological, physical, and social limitations that participants experience as a result of pain. Subscales characterize participants’ physical functioning, role functioning as impacted by physical and emotional health, energy or fatigue, emotional well-being, social functioning, pain, and general health. The 36-item Short Form Health Survey demonstrates excellent strong psychometric properties and predictive validity in a variety of diverse samples, including those experiencing chronic pain [71].

### Exploratory Variables

Demographics, class attendance or home practice, expectancies, and substance use will be measured to examine the impact on the primary and secondary outcomes. A brief questionnaire (minimum data set as recommended by the National Institutes of Health Taskforce on Research Standards for CLBP) [54] will assess demographics (age, gender, race or ethnicity, education, and employment status) and medical or psychiatric history (comorbidities, medication usage, and ongoing treatments). Attendance at yoga or stretching sessions will be assessed using attendance data from the session recordings. The Essential Properties of Yoga Questionnaire [72] will be used to rate both interventions (yoga and stretching or strengthening) and measures the extent to which 14 different dimensions are emphasized in a mind-body intervention (eg, physical challenge, breathwork, restorative postures, and mindfulness). The Credibility Expectancy Questionnaire [73], a 6-item self-report instrument, will be used to assess treatment credibility and patient expectancy for improvement [74]. In order to assess participants’ use of tobacco, alcohol, prescription medicines, cannabis, and other nonprescribed substances, the Alcohol, Smoking, and Substance Involvement Screening Test [75] will be administered by study staff at each time point.

### Statistical Analysis

The primary aim of this study will test the null hypothesis that the 2 population means are equal between the yoga and stretching and strengthening groups on the primary outcome, pain severity as measured by the BPI-SF, as well as the primary MOA, ER as assessed by the DERS. Power calculations incorporated prior effect sizes from works by others. A proposed sample size of 102 per group, which adjusted for attrition, was calculated to detect statistically significant results with 80% power (estimated *d*=0.50). In consideration of our secondary aims, we also powered the detection of paths a and b within pain interference mediation models. This was done using simulation in R (R Foundation for Statistical Computing; N=5000). Following simulation methods (N=5000) presented in Fritz and MacKinnon [76], sample size was determined by increasing simulation sample size until 90% of simulations significantly detected both paths a and b with estimated effect size magnitudes of *d*=0.39 (path a) and *d*=0.26 (path b) at α=.05. A proposed sample size of 116 was calculated, which was already reached when powering our primary aim.

### Data Analysis

All analyses will be performed in SPSS (version 29; IBM Corp). A *P* value of .05 or less will be considered statistically significant. The primary outcome is pain severity (BPI-SF) and the primary MOA is ER (DERS). We will examine baseline demographics and study variables descriptively and test for differences between yoga and stretching and strengthening groups. If assumptions are met, a series of 2-sided *t* tests, mixed effects models, and path analyses will test our hypotheses. A mediation path analysis will be conducted examining the indirect path of group and change in ER from baseline to 6 weeks (path a), and change in ER from baseline to 6 weeks and pain severity (BPI-SF) at 12 weeks (path b) controlling for baseline pain severity. The magnitude of indirect path b will be assessed controlling for path a and the direct path between group and change in pain severity at 12 weeks (path c) to test if changes in ER predict subsequent changes in pain severity. Identical models will be created with change in ER from baseline to 12 weeks and pain severity at 3- and 6-month follow-ups. The magnitude of indirect path b will be compared between groups to test if the path between ER and pain severity is greater in the yoga group than in the stretching and strengthening group. If 95% CIs of path estimates do not contain 0, they will be considered meaningful. Finally, a series of moderated mediation path analyses will be conducted, examining the measures of baseline pain sensitivity (QST scores and normalized gene expression values), strength and flexibility, pain interference, and quality of life as moderators of the identical mediation model. All analyses will also be adjusted for important covariates including, but not limited to demographics (eg, biological sex, race, and sex), cohort, instructor, and intervention components (Essential Properties of Yoga Questionnaire) and credibility or perception of success (Credibility Expectancy Questionnaire). Sensitivity analyses will examine whether treatment responders and nonresponders differ significantly in baseline ER abilities. Baseline DERS scores will also be examined as a continuous moderator in main study analyses to establish whether some individuals may enjoy greater benefit from this intervention more than others.

## Results

This project is funded by the National Center for Complementary and Integrative Health and reviewed by the University of Connecticut’s IRB. Recruitment began in September 2020. As of January 2024, we have enrolled 138 participants. We expect the study to be completed by May 2025. No interim analyses are planned for evaluation of the primary and secondary outcomes. Analyses will be conducted following final data collection using a mediation path analysis and series of moderated mediation path analyses to examine the mechanisms through which yoga reduces pain.

## Discussion

Yoga has been shown to decrease pain severity in individuals with CLBP. This study aims to determine whether ER is a mechanism of yoga through which a significant reduction in pain can be achieved. We will also determine whether yoga can lead to improvements in pain sensitivity, physical strength and flexibility, pain interference, and quality of life.

This will be the first study to assess ER as an MOA of yoga and interrogate plausible biological and psychological pathways that impact the contribution of ER on pain relief. The study results will provide empirical data on the role of ER as a MOA for yoga interventions and inform future studies on whether ER could be bolstered as a component of yoga or other mind-body interventions to improve pain outcomes.

Limitations of the study include that it will be performed at 1 site within the United States, and the interventions are only delivered in English. As a first step to determine whether ER is a MOA of yoga, the study may support evaluating or targeting ER in yoga interventions among more diverse populations, with delivery in other languages, and with other chronic pain conditions.

This 2-arm parallel group blinded RCT will examine ER as the MOA of yoga in individuals with CLBP. The study will provide important data for evaluating whether improvements in ER are responsible for reduced pain, whether pain sensitization moderates yoga’s effects on pain, or if yoga and improved ER abilities reduce pain sensitivity and thereby relieves pain. The study findings have important implications for future yoga research studies, as well as other mind-body modalities designed to improve pain management.

## Appendix

Multimedia Appendix 1 Peer-review report by the National Center for Complementary and Integrative Health (National Institutes of Health).

## Abbreviations

BPI-SF: Brief Pain Inventory-Short Form
CLBP: chronic low back pain
DERS: Difficulties in Emotion Regulation Scale
ER: emotion regulation
IRB: institutional review board
MOA: mechanism of action
QST: quantitative sensory testing
RCT: randomized controlled trial
REDCap: Research Electronic Data Capture

## Data Availability

Data sharing is not applicable to this article as no data sets were generated or analyzed during this study.
