# Supplementary material for: Testing Biological and Psychological Pathways of Emotion Regulation as a Primary Mechanism of Action in Yoga Interventions for Chronic Low Back Pain: Protocol for a Randomized Controlled Trial
Source: JMIR Res Protoc. 2024 Mar 14;13:e56016. doi: 10.2196/56016 (PMC10979342; doi:10.2196/56016)
Supplement: Multimedia Appendix 1 [file resprot_v13i1e56016_app1.pdf]

SUMMARY STATEMENT

PROGRAM CONTACT:
Dr. Inna Belfer
301-435-1573
inna.belfer@nih.gov

(Privileged Communication)

Release Date: **10/21/2019**
Revised Date:

Application Number: **1 R01 AT010555-01A1**

Principal Investigators (Listed Alphabetically):

PARK, CRYSTAL L (Contact)
STARKWEATHER, ANGELA RENEE

Applicant Organization: UNIVERSITY OF CONNECTICUT STORRS

Review Group: **BMIO**
Behavioral Medicine, Interventions and Outcomes Study Section

Meeting Date: **10/03/2019**
Council: **JAN 2020**
Requested Start: **04/01/2020**
RFA/PA: **PA18-323**
PCC: **BELFERI**

Project Title: **Emotion regulation as a primary mechanism of action in yoga interventions for chronic low back pain: An RCT testing biological and psychological pathways**
SRG Action: **Impact Score:20 Percentile:3**
Next Steps: **Visit https://grants.nih.gov/grants/next_steps.htm**
Human Subjects: 30-Human subjects involved - Certified, no SRG concerns
Animal Subjects: 10-No live vertebrate animals involved for competing appl.
Gender: 1A-Both genders, scientifically acceptable
Minority: 1A-Minorities and non-minorities, scientifically acceptable
Age: 1A-Children, Adults, Older Adults, scientifically acceptable

Project Year	Direct Costs Requested	Estimated Total Cost
1	352,979	559,874
2	355,342	563,622
3	432,286	685,666
4	434,958	689,904
5	434,383	688,992
TOTAL	2,009,948	3,188,057

ADMINISTRATIVE BUDGET NOTE: The budget shown is the requested budget and has not been adjusted to reflect any recommendations made by reviewers. If an award is planned, the costs will be calculated by Institute grants management staff based on the recommendations outlined below in the COMMITTEE BUDGET RECOMMENDATIONS section.

1R01AT010555-01A1 Park, Crystal

RESUME AND SUMMARY OF DISCUSSION: This application proposes to test whether yoga improves chronic low back pain (CLBP) by enhancing emotion regulation (ER) and will evaluate whether reduced pain sensitization mediates the pain-reducing effects of yoga-induced improved ER. The study was considered innovative in testing ER as a mediator of yoga's pain-reducing effects. Both the environment and investigators are excellent and were very responsive to prior critiques. Rigor was noted by: use of RCT; well-developed yoga intervention; adequate control condition; strong recruitment and retention plan; use of blinding; inclusion of an intermediate and long-term assessments; and a validated battery for assessment of pain sensitization. There were very few weaknesses noted. In summary, the committee concluded that the potential overall impact was very high.

DESCRIPTION (provided by applicant): Yoga has been shown to have consistent but modest effects in reducing pain and improving function in populations with chronic low back pain (CLBP) in multiple randomized controlled trials. Reviews and practice guidelines support yoga as an evidence-based treatment for CLBP with at least moderate benefit and yoga is being increasingly applied as an integrative therapy. However, the mechanisms through which yoga exerts clinical improvements on pain severity and interference have not been identified; such identification might lead to optimizing yoga interventions to improve their potency. To identify underlying mechanisms associated with yoga interventions (PA 18-323) and based on a comprehensive theoretical emotion regulation model of yoga developed by the PI, we aim to test emotion regulation as a primary mechanism of yoga's effects, and to test biological pathways through which yoga's effects on increased adaptive emotion regulation may operate to affect pain-related functioning. Emotions strongly influence perceptions of pain intensity and predict disability, particularly among individuals with CLBP, and interventions that promote emotion regulation skills have been shown to reduce pain. Increasing evidence demonstrates that consistent yoga practice can promote improved emotion regulation, but research has not yet tested whether the effects of yoga practice on CLBP are due to improvements in emotion regulation. To examine this issue, we will conduct a randomized controlled trial of 204 people with CLBP assessed pre-intervention, 6 weeks, post-intervention and at 3- and 6-month follow-up to test the impact of a yoga intervention on emotion regulation relative to stretching/strengthening without meditation or breathwork. Secondarily, we will also assess whether yoga's effects on CLBP (improved pain severity and functioning) are mediated by changes in emotion regulation. We also examine whether the link between emotion regulation and pain severity and functioning is moderated or mediated by pain sensitization, effects that have been theorized and/or demonstrated to account for changes in pain, thereby testing emotion regulation as a key mechanism underlying the clinical effects of yoga on CLBP.

PUBLIC HEALTH RELEVANCE: By identifying specific mechanisms through which yoga influences pain, the proposed study will advance the scientific study of yoga for multiple pain-related conditions, strengthen the evidence base on yoga for CLBP (facilitating healthcare providers' confidence and likelihood in prescribing yoga), and provide specific directions for optimizing yoga to increase its effectiveness for treating CLBP, one of the most prevalent and costly health conditions in the US.

CRITIQUE 1

Significance: 2
Investigator(s): 1
Innovation: 1
Approach: 2
Environment: 1

Overall Impact: This revised R01 application investigates the mechanisms of yoga as a pain-relieving treatment, via emotional regulation. In 204 people with chronic low back pain, a 12-week yoga intervention is compared with a stretching/strengthening intervention to assess impact on emotional regulation (primary aim), and on pain sensitivity/interference (secondary aim). A second, secondary aim examines the interaction of ER and pain sensitivity/interference. Overall the proposal has very well-explained and rational Significance, Innovation, and a strong Approach with research strategy having robust design likely to yield clinically impactful results. The environment is superior and supporting materials are well-prepared. The proposal is viewed as having high scientific impact.

1. Significance:

Strengths

- A better understanding of the mechanism by which yoga reduces chronic pain would allow for refinement of yoga practices intended as nonpharmacologic pain treatment, focusing on those mechanisms. This might reduce the variability in pain treatment response and make the treatment more viable for broad application, improving health, quality of life, and public health burden.
- Defining yoga mechanisms for low back pain amelioration is also exciting because action via emotional regulation might also help identify patients unlikely to respond, for example patients with apathy.

Weaknesses

- Improved application of yoga as a pain treatment still does not address implementation barriers such as nonadherence, lack of buy-in by third party stakeholders, and shortage of practitioner-teachers.

2. Investigator(s):

Strengths

- The PIs are experienced investigators. Dr. Park has 2 past R01s and 3 synergistic multi-year funded projects. She is a clinical psychologist who has developed research emphasis in emotional self-regulation and also in yoga, thus this project is the natural outgrowth of her body of work in both areas. Dr. Starkweather is a clinician researcher in nursing and psychosocial stress.
- Dr. Finklestein-Fox is also specialized in psychosocial stress and is a clinical psychologist; Dr. Huedo-Medina supports statistical analysis; Dr. Groessl dds further expertise in clinical psychology and yoga clinical trials. Jie Chen is the graduate student supporting the study.

Weaknesses

- None noted.

3. Innovation:

Strengths

- Dissociating fundamental components (key properties/mechanisms) of yoga is highly novel.
- Addressing emotional regulation as a mediator of chronic low back pain is mildly novel.

Weaknesses

- None noted.

4. Approach:

Strengths

- The rigor of past scientific studies is demonstrated in Dr. Park's past work on a self-regulatory model of yoga's intrinsic actions.
- Further supporting the past scientific study rigor are four other studies reviewed, including two pilot studies performed by Dr Park and colleagues and a study by Co-I Groessel consistent with the study purpose, showing improved emotional self-management and reduced pain, including reduced use of opioid medications with yoga in Groessel's study
- The rigor of the current study method is supported by randomized assignment to treatment or control conditions; subjects will be recruited continuously and run in cohorts of 34 to help control for seasonal confounds to effects.
- The active control is designed to stimulate the same muscle groups without relying upon the core components of yoga as identified in the PI's model. Its use is validated by previous yoga studies in which it was used as a comparison condition.
- The study examines changes in gene expression as a biological biomarker of stress and inflammatory changes.

Weaknesses

- The investigators themselves note that this project privileges internal validity in its translational aim of clarifying mechanism for yoga pain reduction. This is true in that pain sensitivity and interference are definitely not equivalent to an adverse experience of pain, or limitations in social participation because of pain.
- For recruitment, we are not given numbers in the Form E section.

5. Environment:

Strengths

- U Conn provides a strong environment for the study. Clinical resources seem adequate; the recruitment and retention plan lists. Thus, it is difficult to know whether the resources provided for recruitment are satisfactory.

Weaknesses

- None noted.

Study Timeline:

Strengths

- Very detailed; informative; excellent.

Weaknesses

Protections for Human Subjects:

Acceptable Risks and/or Adequate Protections

- Acceptable: disclosure and injury.

Data and Safety Monitoring Plan (Applicable for Clinical Trials Only):

Acceptable

- Will assemble a DSMB.

Inclusion Plans:

- Sex/Gender: Distribution justified scientifically
- Race/Ethnicity: Distribution justified scientifically
- For NIH-Defined Phase III trials, Plans for valid design and analysis: Not applicable
- Inclusion/Exclusion Based on Age: Distribution justified scientifically
- 50% women.
- 16% Black/AA, 1% Native, 6% Asian, 3% more than one race and 19% Hispanic/Latinex. These estimates are based on population demographics. An excellent racial and cultural outreach plan is provided.
- Age 18 through [no age limit], satisfactory for age-inclusion.

Vertebrate Animals:

Not Applicable (No Vertebrate Animals)

Biohazards:

Not Applicable (No Biohazards)

Resubmission:

- This revised application is highly responsive to the previous reviewer critique. Among the many changes which strengthened the application are improved clarity of the reason why mechanistic investigation is the primary goal of the study, better description of the role of the PI and team in past research demonstrating the rigor of this scientific line of thinking, clarification of relationships among the study team, a more detailed analysis plan including sex as a biological variable, and a well-justified sample size estimation. An additional follow-up point at 6 weeks has been added to be responsive to reviewer critique.

Resource Sharing Plans:

Acceptable

- Both data on pain and emotional regulation, and data on RNA sequencing, will be made available through data use agreements and manuscripts.

Authentication of Key Biological and/or Chemical Resources:

Acceptable

- Describes 4 key practices to authenticate resources.

Budget and Period of Support:

Recommend as Requested

CRITIQUE 2

Significance: 3
Investigator(s): 1
Innovation: 2
Approach: 2
Environment: 1

Overall Impact: The primary goal of this resubmitted R01 proposal is to test the effects of yoga (vs. stretching/strengthening) on emotion regulation (ER), a potential mechanism of action in the effects of yoga on pain intensity/interference. Secondary aims will focus on examining whether yoga-related improvements in ER reduce pain severity/interference and evaluating the role of pain sensitization as a moderator (Model 1) or mediator (Model 2) of these effects. Participants will be 204 individuals with chronic low back pain (CLBP) who are yoga-naïve and recruited from the local community. Interventions will be conducted with 6 cohorts of patients (n = 34 per cohort), while simultaneously running a stretching and a yoga group (n = 17 each). Outcomes will be assessed at baseline, mid-intervention (6 weeks), end of interventions, and at 3- and 6-month post-intervention follow-ups. CLBP is a significant and costly public health problem, and although yoga is already widely used in the context of pain, mechanisms of action remain poorly understood. The investigators were very responsive to previous reviews, and the resubmitted proposal clarifies why emotional regulation is the primary outcome, more thoroughly addresses the rigor (including strengths and limitations) of extant research on yoga and CLBP, better integrates biological measures in the study conceptualization, and adds a mid-intervention assessment (6-wks). There remains, however, some question as to whether results of the proposed research would lead to greater utilization of yoga for pain in clinical practice. The investigative team is collectively very strong, and the scientific environment at the University of Connecticut Health Center should contribute to the likelihood of a successful outcome. Novel aspects include the study of mechanisms - in general (and ER specifically) - in the effects of yoga on pain, though application of yoga to the context of chronic pain is less innovative. Scientifically rigorous aspects of the approach include utilization of a randomized controlled trial and incorporation of QST pain testing and a behavioral laboratory-based assessment of ER. Despite a few concerns in the approach, including a possible missed opportunity to examine opioid use trajectories and apparent failure to account for other substance use, the potential overall impact of this project remains in the high range.

1. Significance:

Strengths

- CLBP is a significant, prevalent, and costly public health problem.
- There is mounting empirical evidence that yoga can improve ER.
- The rigor of the prior work, including strengths and limitations of extant research examining effects of yoga on chronic pain, was adequately addressed.
- If successful, this research may increase understanding of specific mechanisms by which yoga may influence chronic pain intensity/interference.

Weaknesses

- Yoga is already widely used in the context of pain and there is some question as to whether identification of specific mechanisms of action would lead to greater utilization/dissemination in clinical practice.

2. Investigator(s):

Strengths

- The investigative team is collectively very strong with relevant and complementary expertise that should support the proposed research.
- MPIs have a demonstrated record of collaboration on topics relevant to the proposal, including a pilot study to optimize yoga for low back pain.
- The inclusion of a biostatistician with expertise in multi-level analysis, moderation, mediation, and mixed-effects modeling is a strength.

Weaknesses

- No major limitations noted.

3. Innovation:

Strengths

- Examination of mechanisms underlying the effects of yoga is novel.
- No previous work has examined the effects of yoga on ER in the context of chronic pain.

Weaknesses

- Although mechanisms of action remain poorly understood, the application of yoga to pain in general and CLBP in particular is not innovative.

4. Approach:

Strengths

- Scientifically rigorous aspects of the approach include use of a two-arm randomized interventional design, inclusion of QST pain testing, and the selection of a control condition (stretching/strengthening) that can account for nonspecific effects such as time, attention, and effort, along with general improvements in strength and flexibility.
- Preliminary studies examining the effects of yoga for back pain (a well as potential mediators) provide important support for the proposed approach.
- The inclusion of a behavioral laboratory-based assessment of ER (The Negative Affective Priming Task) to complement the primary Difficulties in Emotion Regulation Scale is another notable strength.

Weaknesses

- The application notes that medication is the primary treatment among CLBP patients, and preliminary work conducted by Co-I Dr. Groessl demonstrated a main effect of yoga in terms of decreased use of opioid medications. Although inclusion criteria require that medication use must be stable for 30 days and that participants will be asked to take their lowest usual dose of pain medication within 24 hours of each assessment, there may be a missed opportunity to examine the effects of yoga and ER on pain medication use trajectories.
- It does not appear that concurrent substance use (e.g., nicotine, alcohol, cannabis) will be assessed or accounted for despite evidence that prolonged and acute use of such substances can uniquely influence self-reported pain intensity/interference and reactivity to QST assessment.

5. Environment:

Strengths

- The scientific environment at the University of Connecticut Health Center (UCHC) is strong and should support the proposed research.
- Yoga sessions will take place at the UCHC Outpatient Pavilion building, and a letter of support was provided.

Weaknesses

- No major limitations noted.

Study Timeline:

Strengths

- Study timeline and milestones were described in detail.

Weaknesses

- No major limitations noted.

Protections for Human Subjects:

Acceptable Risks and/or Adequate Protections

- Potential Risks and Adequacy of Protection against risks were described in detail.

Data and Safety Monitoring Plan (Applicable for Clinical Trials Only):

Acceptable

- o An acceptable data and safety monitoring plan were provided. A Data Safety and Monitoring Board will also be assembled.

Inclusion Plans:

- Sex/Gender: Distribution justified scientifically
- Race/Ethnicity: Distribution justified scientifically
- For NIH-Defined Phase III trials, Plans for valid design and analysis: Not applicable
- Inclusion/Exclusion Based on Age: Distribution justified scientifically
- 50% Female, 68% White, 15% Black or African American, 6% Asian, 14% Hispanic or Latino
- men and women age 18+ years of age will be included

Vertebrate Animals:

Not Applicable (No Vertebrate Animals)

Biohazards:

Acceptable

- All study staff will complete Biohazard and Transportation of Biohazard training through UConn Environmental Safety Department. Collection, storage and assays of biohazardous samples will be performed in accordance with established safety guidelines and regulatory requirements.

Resubmission:

- Investigators were very responsive to the previous reviews.

Resource Sharing Plans:

Acceptable

- Data and resource sharing plan were provided.

Authentication of Key Biological and/or Chemical Resources:

Acceptable

- In order to ensure the identity and validity of key biological and/or chemical resources used in RNA sequencing as proposed in this application, the research team has established the following practices: 1. All chemicals and reagents are purchased tracking catalog numbers from well-established companies/vendors; 2. Batch numbers and other detailed product information are recorded in electronic lab notes for reference and future purchase orders; 3. Quality control analyses are performed for all key biological and/or chemical resources that could influence the research data (RNA quality, RIN >7.0); 4. Quality controls are performed for all experiments and reported to the PI. All decisions made by the PI are reported on the raw data output for analysis and reporting of results in research publications and presentations.

Budget and Period of Support:

Recommend as Requested

CRITIQUE 3

Significance: 2
Investigator(s): 1
Innovation: 1
Approach: 3
Environment: 1

Overall Impact: This resubmission application proposes to test whether yoga improves chronic low back pain (CLBP) by enhancing emotion regulation (ER) and will investigate whether reduced pain sensitization mediates the pain-reducing effects of yoga-induced improved ER. The project addresses a condition of tremendous public health impact, as CLBP is the leading cause of disability worldwide and existing treatments are not particularly effective. The rigor of prior research is addressed, and the proposed research will address important knowledge gaps regarding the mechanisms whereby yoga's beneficial effects in CLBP are achieved. The scientific rationale is well-founded in existing literature as captured in a new integrative model of yoga's therapeutic effects developed by this team. One surprising omission in the model is consideration of baseline ER as an important predictor of treatment response. That is, the investigators emphasize that the yoga interventions in prior studies may have had varying impact on ER, which explains the variable intervention effects. However, it is at least as plausible that dysfunctional ER contributes to CLBP in only a proportion of patients, and these patients are most likely to benefit from yoga. Also, the secondary aims are somewhat dependent on Aim 1, though this is not a major concern given prior findings that yoga improves ER. The investigative team is excellent, as the contact PI has strong expertise in clinical intervention studies of yoga and emotion regulation has developed a novel model of yoga's effects on ER. The MPI is a well-established expert in CLBP with valuable experience assessing pain-related biomarkers and pain sensitization. The Co-Is bring important expertise in mind-body interventions and biostatistics. The project is innovative in

testing ER as a mediator of yoga's pain-reducing effects, and the addition of pain sensitization as a mediator and moderator of treatment effect is also novel. The strengths of the approach include: use of a well-developed yoga intervention based on considerable preliminary findings, a viable control condition that mimics many of the nonspecific effects of yoga, a strong recruitment and retention plan, use of blinding to enhance rigor, the inclusion of an intermediate assessment timepoint as well as long-term follow-up, and a validated comprehensive battery for assessment of pain sensitization. Rigor will be enhanced by blinding of assessors, video-based assessment of treatment fidelity, assessment of attendance and home practice, and a manualized yoga intervention to promote standardization. Also, the investigators have been quite responsive to prior critiques. A few minor weaknesses in the approach include: lack of information on management of the multiple variables that will be generated by the quantitative sensory testing protocol, no plan to include baseline ER as a potential treatment moderator, and limited attention to managing concomitant therapies in the analytic plan. The environment at the University of Connecticut is ideally suited for the proposed work. However, these do little to detract from the enthusiasm for this novel and exciting application.

Study Timeline:

Strengths

- The timeline is very detailed, providing a clear and plausible depiction of when study milestones will be accomplished.

Weaknesses

- None noted.

Protections for Human Subjects:

Acceptable Risks and/or Adequate Protections

- The risks of the project are thoroughly discussed, and adequate protections are in place to mitigate risks.

Data and Safety Monitoring Plan (Applicable for Clinical Trials Only):

Acceptable

- The team will establish a DSMB to monitor data and safety.

Inclusion Plans:

- Sex/Gender: Distribution justified scientifically
- Race/Ethnicity: Distribution justified scientifically
- For NIH-Defined Phase III trials, Plans for valid design and analysis: Not applicable
- Inclusion/Exclusion Based on Age: Distribution justified scientifically
- Equal numbers of females and males will be enrolled. Racial/ethnic diversity is modest but consistent with the local population: 58% non-Hispanic white, 14% Black or African American, 14% Hispanic, 6% Asian, 8% other.

Vertebrate Animals:

Not Applicable (No Vertebrate Animals)

Biohazards:

Not Applicable (No Biohazards)

Resubmission:

None noted by reviewer.

Resource Sharing Plans:

Acceptable

- There is a plan to share the genomic data via the NIH Gene Expression Omnibus repository.

Budget and Period of Support:

Recommended budget modifications or possible overlap identified:

- Budget appears appropriate and well-justified.

THE FOLLOWING SECTIONS WERE PREPARED BY THE SCIENTIFIC REVIEW OFFICER TO SUMMARIZE THE OUTCOME OF DISCUSSIONS OF THE REVIEW COMMITTEE, OR REVIEWERS' WRITTEN CRITIQUES, ON THE FOLLOWING ISSUES:

PROTECTION OF HUMAN SUBJECTS: ACCEPTABLE

INCLUSION OF WOMEN PLAN: ACCEPTABLE

INCLUSION OF MINORITIES PLAN: ACCEPTABLE

INCLUSION ACROSS THE LIFESPAN PLAN: ACCEPTABLE

COMMITTEE BUDGET RECOMMENDATIONS: The budget was recommended as requested.

Footnotes for 1 R01 AT010555-01A1; PI Name: Park, Crystal L

NIH has modified its policy regarding the receipt of resubmissions (amended applications). See Guide Notice NOT-OD-14-074 at http://grants.nih.gov/grants/guide/notice-files/NOT-OD-14-074.html. The impact/priority score is calculated after discussion of an application by averaging the overall scores (1-9) given by all voting reviewers on the committee and multiplying by 10. The criterion scores are submitted prior to the meeting by the individual reviewers assigned to an application, and are not discussed specifically at the review meeting or calculated into the overall impact score. Some applications also receive a percentile ranking. For details on the review process, see http://grants.nih.gov/grants/peer_review_process.htm#scoring.

MEETING ROSTER

Behavioral Medicine, Interventions and Outcomes Study Section
Risk, Prevention and Health Behavior Integrated Review Group
CENTER FOR SCIENTIFIC REVIEW
BMIO
10/03/2019 - 10/04/2019

Notice of NIH Policy to All Applicants: Meeting rosters are provided for information purposes only. Applicant investigators and institutional officials must not communicate directly with study section members about an application before or after the review. Failure to observe this policy will create a serious breach of integrity in the peer review process, and may lead to actions outlined in NOT-OD-14-073 at https://grants.nih.gov/grants/guide/notice-files/NOT-OD-14-073.html and NOT-OD-15-106 at https://grants.nih.gov/grants/guide/notice-files/NOT-OD-15-106.html, including removal of the application from immediate review.

CHAIRPERSON(S)

FILLINGIM, ROGER B, PHD
DISTINGUISHED PROFESSOR
DEPARTMENT OF COMMUNITY DENTISTRY
AND BEHAVIORAL SCIENCE
COLLEGE OF DENTISTRY
UNIVERSITY OF FLORIDA
GAINESVILLE, FL 32610

MEMBERS

BARRETT, A M, MD
PROFESSOR
DEPARTMENT OF PHYSICAL MEDICINE
AND REHABILITATION
RUTGERS NEW JERSEY MEDICAL SCHOOL
WEST ORANGE , NJ 07052

BRUEHL, STEPHEN, PHD
PROFESSOR
DEPARTMENT OF ANESTHESIOLOGY
SCHOOL OF MEDICINE
VANDERBILT UNIVERSITY MEDICAL CENTER
NASHVILLE, TN 37212

BURG, MATTHEW M, PHD
PROFESSOR
DEPARTMENT OF INTERNAL MEDICINE
YALE UNIVERSITY SCHOOL OF MEDICINE
NEW HAVEN, CT 06520

BURNS, JOHN W., PHD *
PROFESSOR
DEPARTMENT OF BEHAVIORAL SCIENCES
RUSH UNIVERSITY MEDICAL CENTER
CHICAGO, IL 60612

CHARVET, LEIGH, PHD *
ASSOCIATE PROFESSOR
NEW YORK UNIVERSITY MEDICAL CENTER
NEW YORK, NY 10016

COHEN, RONALD A, PHD *
PROFESSOR AND DIRECTOR
CENTER FOR COGNITIVE AGING AND MEMORY
MCKNIGHT BRAIN INSTITUTE
UNIVERSITY OF FLORIDA
GAINESVILLE, FL 32610

DITRE, JOSEPH W, PHD
ASSOCIATE PROFESSOR
DEPARTMENT OF PSYCHOLOGY
SYRACUSE UNIVERSITY
SYRACUSE, NY 13104

DURANT, RAEGAN W, MD, MPH
ASSOCIATE PROFESSOR
DEPARTMENT OF MEDICINE
DIVISION OF PREVENTIVE MEDICINE
UNIVERSITY OF ALABAMA AT BIRMINGHAM
BIRMINGHAM, AL 35294

ESTABROOKS, PAUL, PHD *
PROFESSOR
DEPARTMENT OF HEALTH PROMOTIONS,
SOCIAL & BEHAVIORAL HEALTH
COLLEGE OF PUBLIC HEALTH
UNIVERSITY OF NEBRASKA MEDICAL CENTER
OMAHA, NE 68198

HASSETT, AFTON L, PSYD *
ASSOCIATE RESEARCH SCIENTIST
DEPARTMENT OF ANESTHESIOLOGY
CHRONIC PAIN AND FATIGUE RESEARCH CENTER
UNIVERSITY OF MICHIGAN MEDICAL SCHOOL
ANN ARBOR, MI 48106

HOULE, TIMOTHY T, PHD *
ASSOCIATE PROFESSOR
ANESTHESIA-MASSACHUSETTS GENERAL HOSPITAL
HARVARD MEDICAL SCHOOL
BOSTON, MA 02114

HUI, DAVID, MD
ASSOCIATE PROFESSOR
DEPARTMENT OF PALLIATIVE CARE AND
REHABILITATION MEDICINE
UNIVERSITY OF TEXAS MD ANDERSON CANCER CENTER
HOUSTON, TX 77030

JENSEN, MARK P, PHD
PROFESSOR AND VICE CHAIR FOR RESEARCH
DEPARTMENT OF REHABILITATION MEDICINE
UNIVERSITY OF WASHINGTON
SEATTLE, WA 98195

KNIGHT, SARA J, PHD
PROFESSOR AND RESEARCH SCIENTIST
DIVISION OF EPIDEMIOLOGY
DEPARTMENT OF INTERNAL MEDICINE
UNIVERSITY OF UTAH
SALT LAKE CITY, UT 84132

LENGACHER, CECILE A, PHD *
PROFESSOR AND DIRECTOR
BS-PHD PROGRAM
COLLEGE OF NURSING
UNIVERSITY OF SOUTH FLORIDA
TAMPA, FL 33612-4766

LENZE, ERIC J, MD
PROFESSOR
DEPARTMENT OF PSYCHIATRY
WASHINGTON UNIVERSITY SCHOOL OF MEDICINE
SAINT LOUIS, MO 63110

LUYSTER, FAITH S, PHD *
ASSISTANT PROFESSOR
SCHOOL OF NURSING
UNIVERSITY OF PITTSBURGH
PITTSBURGH, PA 15261

MA, JUN, MD, PHD *
BETH AND GEORGE VITOUX PROFESSOR OF MEDICINE
DEPARTMENT OF MEDICINE
COLLEGE OF MEDICINE
UNIVERSITY OF ILLINOIS AT CHICAGO
CHICAGO, IL 60608

MATHEW, JOSEPH P, MD, MHSC
PROFESSOR
DEPARTMENT OF ANESTHESIOLOGY
DUKE UNIVERSITY MEDICAL CENTER
DURHAM, NC 27710

PALESH, OXANA G, PHD
ASSOCIATE PROFESSOR
DEPARTMENT OF PSYCHIATRY AND BEHAVIORAL SCIENCES
STANFORD CANCER INSTITUTE
STANFORD UNIVERSITY
STANFORD, CA 94305

PATTON, SUSANA R, PHD
CENTER DIRECTOR
CENTER FOR HEALTHCARE DELIVERY SCIENCE
NEMOURS CHILDRENS HEALTH SYSTEM
JACKSONVILLE, FL 32259

PERRY, TAMARA T, MD
ASSOCIATE PROFESSOR
DEPARTMENT OF PEDIATRICS
DIVISION OF ALLERGY IMMUNOLOGY
UNIVERSTIY OF ARKANSAS FOR MEDICAL SCIENCES
LITTLE ROCK, AR 72202

PEUGH, JAMES, PHD *
RESEARCH ASSOCIATE PROFESSOR
DEPARTMENT OF PEDIATRICS
DIVISIONS OF BEHAVIORAL MEDICINE AND CLINICAL PSYCHOLOGY AND BIOSTATISTICS AND EPIDEMIOLOGY
CINCINNATI CHILDREN'S HOSPITAL AND MEDICAL CENTER
CINCINNATI, OH 45229--302

RINI, CHRISTINE, PHD
PROFESSOR
DEPARTMENT OF MEDICAL SOCIAL SCIENCES
NORTHWESTERN UNIVERSITY FEINBERG SCHOOL OF MEDICINE
CHICAGO, IL 60611

SMITH, PATRICK J, PHD
ASSOCIATE PROFESSOR
DEPARTMENT OF PSYCHIATRY AND BEHAVIORAL MEDICINE
DUKE UNIVERSITY
DURHAM, NC 27710

STEWART, JESSE C, PHD
PROFESSOR
DEPARTMENT OF PSYCHOLOGY
INDIANA UNIVERSITY-PURDUE UNIVERSITY INDIANAPOLIS
INDIANAPOLIS, IN 46202

UEBELACKER, LISA A, PHD
PROFESSOR
PSYCHOSOCIAL RESEARCH
BUTLER HOSPITAL
BROWN UNIVERSITY
PROVIDENCE, RI 02906

WEN, KUANG-YI, PHD *
ASSOCIATE PROFESSOR
DIVISION OF POPULATION SCIENCE
SIDNEY KIMMEL CANCER CENTER
THOMAS JEFFERSON UNIVERSITY
PHILADELPHIA, PA 19107

SCIENTIFIC REVIEW OFFICER

MANN, LEE S, PHD
SCIENTIFIC REVIEW OFFICER
CENTER FOR SCIENTIFIC REVIEW
NATIONAL INSTITUTES OF HEALTH
BETHESDA, MD 20892

EXTRAMURAL SUPPORT ASSISTANT

WATTS, MELISSA
EXTRAMURAL SUPPORT ASSISTANT
CENTER FOR SCIENTIFIC REVIEW
NATIONAL INSTITUTE FOR HEALTH
BETHESDA, MD 20892

* Temporary Member. For grant applications, temporary members may participate in the entire meeting or may review only selected applications as needed.

Consultants are required to absent themselves from the room during the review of any application if their presence would constitute or appear to constitute a conflict of interest.
